# miR‐195‐3p alleviates homocysteine‐mediated atherosclerosis by targeting IL‐31 through its epigenetics modifications

**DOI:** 10.1111/acel.13485

**Published:** 2021-09-30

**Authors:** Jiantuan Xiong, Fang Ma, Ning Ding, Lingbo Xu, Shengchao Ma, Anning Yang, Yinju Hao, Huiping Zhang, Yideng Jiang

**Affiliations:** ^1^ School of Basic Medical Sciences Ningxia Medical University Yinchuan China; ^2^ NHC Key Laboratory of Metabolic Cardiovascular Diseases Research Ningxia Medical University Yinchuan China; ^3^ Ningxia Key Laboratory of Vascular Injury and Repair Research Ningxia Medical University Yinchuan China; ^4^ Prenatal Diagnosis Center, General Hospital of Ningxia Medical University Yinchuan China

**Keywords:** DNA methylation, H3K9 acetylation, homocysteine, inflammation, miR‐195‐3p

## Abstract

Atherosclerosis is a serious age‐related disease, which has a tremendous impact on health care globally. Macrophage inflammation is crucial for the initiation and progression of atherosclerosis, and microRNAs (miRNAs) recently have emerged as potent modulators of inflammation, while the underlying mechanisms of its involvement in homocysteine (Hcy)‐mediated macrophage inflammation of atherosclerosis remain largely unknown. Here, we demonstrated that elevated Hcy inhibits the expression of miR‐195‐3p, which in turn enhances IL‐31 expression and thereby causes the secretion of macrophages pro‐inflammatory factors IL‐1β, IL‐6 and TNF‐α and accelerate atherosclerosis. Furthermore, we identified that Hcy can induce DNA hypermethylation and H3K9 deacetylation of miR‐195‐3p promoter due to the increased the binding of DNMT3a and HDAC11 at its promoter. More importantly, Sp1 interacts with DNMT3a suppressed the binding of HDAC11 at miR‐195‐3p promoter and promoted its transcription. In summary, our results revealed a novel mechanism that transcriptional and epigenetic regulation of miR‐195‐3p inhibits macrophage inflammation through targeting IL‐31, which provides a candidate diagnostic marker and novel therapeutic target in cardiovascular diseases induced by Hcy.

## Graphical abstract

Proposed regulation model of miR‐195‐3p expression in Hcy‐induced macrophage inflammation during the formation of atherosclerotic plaque. Downregulation of miR‐195‐3p accelerated Hcy‐induced pro‐inflammation factor expression in macrophage and formation of plaques in *ApoE^−/−^* mice by targeting IL‐31, DNMT3a and HDAC11 are responsible for the downregulation of miR‐195‐3p, and Sp1 interacts with DNMT3a was essential for HDAC11 to bind to miR‐195‐3p promoter.

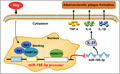

## 1 INTRODUCTION

With the increase of an aging population, aging‐related cardiovascular diseases confer a heavy economic burden on society. The incidence of atherosclerosis increases dramatically with advancing age and constitutes the main cause of cardiovascular diseases mortality and morbidity in the elderly (Hopkins, 2013). Atherosclerosis is a chronic progressive inflammatory disease, recent insights into the pathogenesis of atherosclerosis emphasize the importance of vascular inflammation in the initiation and development of atherosclerotic plaque (Wolf & Ley, 2019). Homocysteine (Hcy) is well recognized to be an independent risk factor for atherosclerosis, in which macrophages play essential roles in the development of atherosclerosis through formation of foam cells and production of inflammatory factors. However, the mechanisms involving in Hcy‐related inflammation in macrophages during atherosclerotic plaque formation is still a matter of debate (McCully, 2015b; Wang et al., 2017).

It has been widely accepted that aberrant expression of genes and cytokines contribute to the onset of atherosclerosis, and the focus on the origin and progression of atherosclerosis has shifted from genetic to epigenetic regulation in recent studies (Kuznetsova et al., 2020; Rizzacasa et al., 2019). microRNAs (miRNAs) are a kind of single‐stranded endogenous small non‐coding RNAs containing 19 to 25 nucleotides and has been now recognized as one of the major epigenetic modulators (Lu & Rothenberg, 2018). There is growing evidence that miRNAs could be able to regulate atherosclerosis physiological and pathological processes through destabilizing target mRNAs and/or inhibiting translation. For example, miR‐19b promotes the secretion of inflammatory cytokines and macrophages intrusion into the endothelial layer, promoting the progression of atherosclerotic lesion (Li et al., 2018). In addition, miR‐181b decreases atherosclerotic plaque formation through upregulation of macrophages TIMP‐3 expression, meaning miR‐181b therapy may serve as another atheroprotective therapeutic strategy (Di Gregoli et al., 2017). These findings suggest that exploring miRNAs effects on atherosclerosis may allow us to exploit them as novel therapeutics or clinical biomarkers thus lead to better management of atherosclerosis.

Of note, as a part of epigenetic machinery, miRNAs are also epigenetically modified by DNA methylation and histone modification like any other protein‐coding genes (Yao et al., 2019). Our previous study revealed that DNMT3b‐mediated hypomethylation of miR‐30a exerts a protection effect of hypoxia postconditioning on aged cardiomyocytes hypoxia/reoxygenation injury (Wang et al., 2020). Hcy is an important intermediate metabolite in the methionine cycle, which has been reported to alter DNA methyltransferase activity, and influence histone deacetylase through the interference of methyl group transferring metabolism (Jakubowski, 2019; Li et al., 2017). As a part of the epigenome, studies have shown that Hcy induces cardiac hypertrophy by promoting MEF2C‐HDAC1 complex formation (Kesherwani et al., 2015). Numerous studies demonstrate that in addition to epigenetic mechanisms, regulatory proteins (transcription factors) also exert an essential and fundamental effect on the transcriptional regulation of specific gene or miRNA (Kim et al., 2019). Sp1 is a sequence‐specific transcription factor, which binds to the GC and GT boxes to initiate and activate a wide range of viral and cellular genes transcription (Vizcaíno et al., 2015). Sp1 could inhibit the DNA methylation of POLD1 gene promoter by binding and the suppressing DNMT1 activities in breast cancer (Zhang et al., 2016). Eukaryotes express genes in incredible diversity manner, which is largely dependent on the interaction between transcription factors and epigenetic regulation. For instance, Sp1 could also recruit repressor complexes, such as the HDAC1‐HDAC2‐mSin3a complex to repress gene transcription (Zhang & Dufau, 2003).

Herein, we identified miR‐195‐3p as a novel miRNA which was associated with macrophage inflammation and atherosclerosis induced by Hcy. Mechanistically, Sp1 interacts with DNMT3a to suppress HDAC11 binding to miR‐195‐3p promoter to increase its expression. Our findings shed new light on the involvement of miR‐195‐3p in macrophage inflammation, which might provide a potential therapeutic strategy for atherosclerosis induced by Hcy.

## 2 RESULTS

### 2.1 Homocysteine accelerates macrophage inflammation during atherosclerotic plaque formation

**FIGURE 1:**
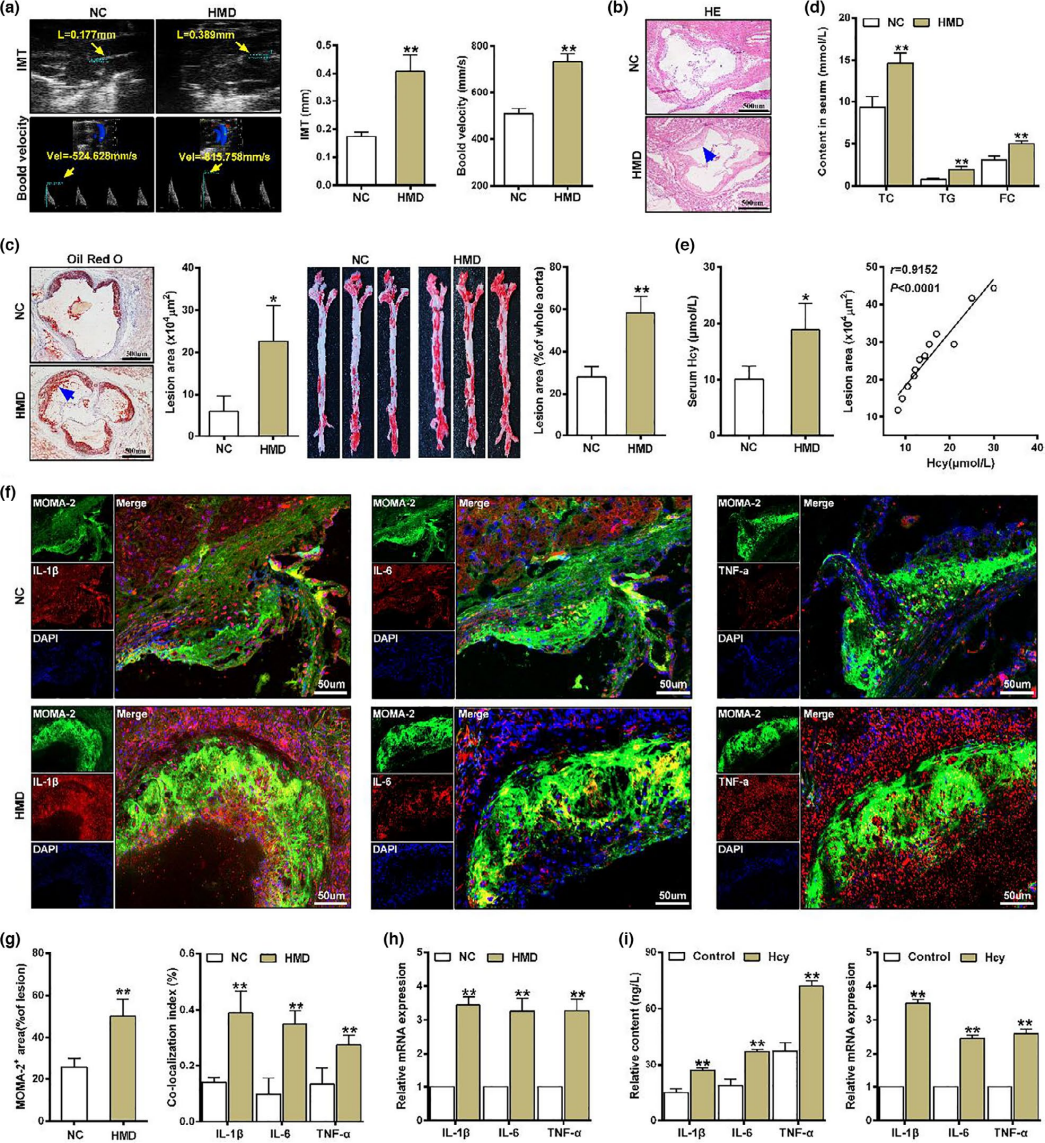
Homocysteine promotes macrophage inflammation and atherosclerosis in *ApoE* ^−/−^ mice. After the *ApoE* ^−/−^ mice were fed with regular diet (NC) or high methionine diet (HMD) for 16 weeks (*n* = 6), then (a) Representative images and quantification of the intima‐media thickness (IMT, yellow arrow) in the aortic root and blood velocity (yellow arrow) in the ascending aorta by ultrasound biomicroscopy (UBM). (b) Photomicrographs of the aortic sinus cross‐sections staining with HE staining. The blue arrow indicates the plaque region. Scale bar =500 μm. (c) Representative images of Oil Red O staining of the aortic sinus cross‐sections (the blue arrow indicates the plaque region, Scale bar =500 μm) and en face aortas and quantitative analysis of the percentage of atherosclerotic plaque area from *ApoE* ^−/−^ mice. (d) The serum levels of total cholesterol (TC), triglyceride (TG) and free cholesterol (FC) in *ApoE* ^−/−^ mice were measured by automatic biochemistry analyzer. (e) Serum levels of Hcy in *ApoE* ^−/−^ mice were measured by automatic biochemistry analyzer, and the correlation between atherosclerotic plaque area and serum Hcy levels was evaluated by Pearson correlation analysis. (f, g) Representative immunofluorescence images and quantification of IL‐1β, IL‐6, and TNF‐α (red) co‐localization with MOMA‐2 (a marker for macrophages, green) in *ApoE* ^−/−^ mice. Nuclei were stained with DAPI (blue). Scale bar =50 μm. (h) The mRNA expression of IL‐1β, IL‐6 and TNF‐α in the aortic from *ApoE* ^−/−^ mice were assessed by qRT‐PCR. (i) The IL‐1β, IL‐6, and TNF‐α secretion in supernatant and mRNA expression in macrophages treated with 100 μmol/L Hcy were detected by ELISA and qRT‐PCR, respectively. Data were presented as mean ±SD. *p < 0.05, **p < 0.01

To get a better insight into the possible mechanism of atherosclerosis induced by Hcy, the *ApoE* ^−/−^ mice were fed with regular diet (NC) and high methionine diet (HMD) as described in experimental procedures. An elevation of the intima‐media thickness (IMT) of aortic root and increase of blood flow velocity of the ascending aortic were observed from HMD‐fed *ApoE* ^−/−^ mice by ultrasound biomicroscopy (UBM) (Figure 1a). HE and Oil Red O staining showed that atherosclerotic lesions of aortic root and whole aorta were remarkably increased in HMD‐fed *ApoE* ^−/−^ mice (Figure 1b,c); consistent with above, the serum levels of total cholesterol (TC), triglyceride (TG) and free cholesterol (FC) were highlighted in HMD‐fed *ApoE* ^−/−^ mice compared with NC‐fed *ApoE* ^−/−^ mice (Figure 1d). Particularly, the serum levels of Hcy were also increased, which was positively correlated with atherosclerotic plaque area (Figure 1e). These results confirmed that Hcy exacerbated atherosclerosis in *ApoE* ^−/−^ mice.

Atherosclerosis is a chronic inflammatory disease and recent study indicated that atherosclerosis‐related inflammation is mainly mediated by pro‐inflammatory cytokines (IL‐1β, IL‐6 and TNF‐α) and inflammatory signaling pathways (Gisterå & Hansson, 2017). To explore the role of inflammation in Hcy‐induced atherosclerosis, the levels of pro‐inflammatory cytokines IL‐1β, IL‐6 and TNF‐α were validated by immunofluorescence staining analysis. The number of IL‐1β, IL‐6 and TNF‐α puncta in atherosclerotic plaque of the HMD‐fed *ApoE* ^−/−^ mice were significantly increased, which was evidenced by the increased co‐localization with MOMA‐2 in aortic root (Figure 1f,g). Meanwhile, a remarkably increase of mRNA expression of IL‐1β, IL‐6 and TNF‐α were observed in HMD‐fed *ApoE* ^−/−^ mice (Figure 1h). In addition, the results also showed that the secretion of IL‐1β, IL‐6 and TNF‐α in supernatant of macrophages were increased after Hcy treatment, as well as the mRNA expression of IL‐1β, IL‐6 and TNF‐α (Figure 1i). Together, these data demonstrated that Hcy facilitated macrophage inflammation in atherosclerosis in *ApoE* ^−/−^ mice.

### 2.2 miR‐195‐3p attenuates inflammation and atherosclerotic plaque formation by targeting IL‐31

**FIGURE 2:**
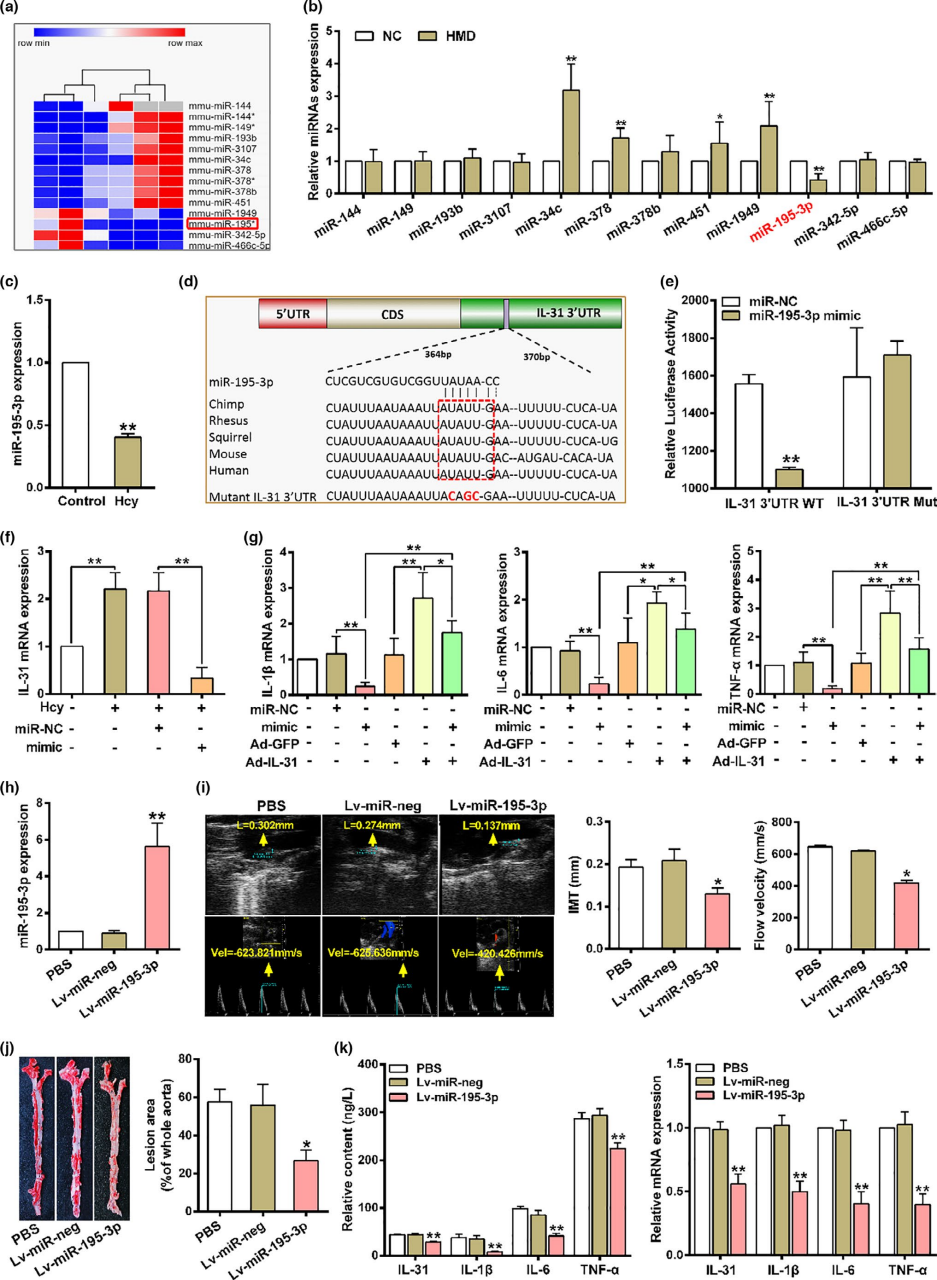
miR‐195‐3p protects against atherosclerosis in *ApoE* ^−/−^ mice by targeting IL‐31 to exert anti‐inflammatory effect. (a) The heatmap shows differential expression of miRNAs in the aorta of *ApoE* ^−/−^ mice with fold change≥2.0, *p* < 0.05. The expression values are represented by a color scale, red indicated high relative expression, blue indicated low relative expression, and white indicated no change. (b) Differential expressed miRNAs (miR‐144, miR‐149, miR‐193b, miR‐3107, miR‐34c, miR‐378, miR‐378b, miR‐451, miR‐1949, miR‐195‐3p, miR‐342‐5p, miR‐466c‐5p) from Microarray analysis data were confirmed using qRT‐PCR in the aortic from *ApoE* ^−/−^ mice fed with regular diet (NC) or high methionine diet (HMD) (*n* = 6). (c) The expression of miR‐195‐3p in macrophages treated with 100 μmol/L Hcy was determined by qRT‐PCR. (d) The miR‐195‐3p binding site in the 3'‐UTR region of IL‐31 was highly conserved among several species. The red box indicated the conserved sequence of IL‐31 via targeting to miR‐195‐3p, and the red letters showed the mutation of the miR‐195‐3p target in IL‐31. (e) The reporter constructs containing 3'‐UTR regions of the wild‐type (WT) and mutant‐type (Mut) IL‐31 were co‐transfected with miRNA negative control (miR‐NC) or miR‐195‐3p mimic. Relative luciferase activities are normalized by the ratio of firefly and renilla luciferase activities. (f) The expression of IL‐31 was detected by qRT‐PCR in macrophages transfected with miR‐NC or miR‐195‐3p mimic. (g) The mRNA expression of IL‐1β, IL‐6 and TNF‐α were detected in macrophages transfected with miR‐NC, miR‐195‐3p mimic, adenovirus expressing IL‐31 (Ad‐IL‐31) and Ad‐GFP solely or collectively in presence of Hcy. (h–j) The expression of miR‐195‐3p, IMT (yellow arrow) of the aortic root, blood velocity (yellow arrow) of the ascending aorta, and atherosclerosis lesion area were detected by qRT‐PCR, UBM and Oil Red O staining after the HMD‐fed *ApoE* ^−/−^ mice were injected with PBS, Lv‐miR‐neg and Lv‐miR‐195‐3p. (k) IL‐31, IL‐1β, IL‐6 and TNF‐α contents in serum and mRNA levels in the aorta from HMD‐fed *ApoE* ^−/−^ mice injected with PBS, Lv‐miR‐neg and Lv‐miR‐195‐3p were measured by ELISA and qRT‐PCR, respectively. Data were presented as mean ±SD. *p < 0.05, **p < 0.01

To screen miRNAs that are involved in inflammation and atherosclerosis induced by Hcy, we assessed changes of miRNA expression in the aorta from HMD‐fed *ApoE* ^−/−^ mice. Hierarchical clustering of miRNA expression showed 12 differentially expressed miRNAs in the aorta of HMD‐fed *ApoE* ^−/−^ mice (Figure 2a). Among them, only miR‐195‐3p showed the most remarkable decreased, and a similar decrease of miR‐195‐3p expression was also observed in macrophages treated with Hcy (Figure 2b,c). Since the discovery of miRNA regulation of genes, several studies have been focused on predicting the biologically relevant target genes for miRNAs. The bioinformatics analyses showed that IL‐31 is the most potential target for miR‐195‐3p to exert inflammatory function (Figure S1a). Computational miRNA‐targeted analysis further found the binding sites of miR‐195‐3p and IL‐31 were highly conserved (Figure 2d). Then, we utilized a pGL3‐IL‐31–3'‐UTR luciferase reporter vector to check if the sequence were in response to miR‐195‐3p. Co‐transfection of miR‐195‐3p mimic with IL‐31–3'‐UTR reporter (WT) in HEK293 cells diminished the relative luciferase activity, while the reporter gene vector containing mutation predicted sequences of IL‐31 (pGL3‐IL‐31‐mut‐3'‐UTR, Mut) had no response to miR‐195‐3p mimic (Figure 2e). Meanwhile, a remarkably increase of IL‐31 expression was observed in HMD‐fed *ApoE* ^−/−^ mice (Figure S1b,c). Next, we transfected miRNA negative control (miR‐NC) and miR‐195‐3p mimic into macrophages (Figure S1d). qRT‐PCR results showed that upregulation of miR‐195‐3p reduced the mRNA expression of IL‐31 (Figure 2f). To further determine the function of miR‐195‐3p in Hcy‐induced macrophage inflammation by targeting IL‐31, miR‐195‐3p mimic and the adenovirus expressing IL‐31 (Ad‐IL‐31) was co‐transfected into macrophages (Figure S1e). As shown in Figure 2g, overexpression of miR‐195‐3p inhibited the mRNA expression of IL‐1β, IL‐6 and TNF‐α in macrophages treated with Hcy, which can be abolished by overexpressed IL‐31. Meanwhile, we observed similar results in the secretion of IL‐31, IL‐1β, IL‐6 and TNF‐α of macrophages supernatant after Hcy treatment (Figure S1f), indicating miR‐195‐3p could inhibit Hcy‐induced macrophage inflammation through IL‐31. These outcomes exhibited that the miR‐195‐3p binding sequence in the 3'‐UTR of IL‐31 is required for miR‐195‐3p‐mediated suppression of macrophage inflammation.

Based on the above results, the lentiviral vector expressing miR‐195‐3p was injected into HMD‐fed *ApoE* ^−/−^ mice through tail vein. Notably, in situ hybridization analysis revealed a significant accumulation of miR‐195‐3p in MOMA‐2‐positive areas of atherosclerotic plaques (Figure S1g,h), and miR‐195‐3p expression was increased in the aortic of HMD‐fed *ApoE* ^−/−^ mice (Figure 2h). Meanwhile, IMT of the aortic root and blood velocity of the ascending aorta were significantly decreased in HMD‐fed *ApoE* ^−/−^ mice injected with Lv‐miR‐195‐3p (Figure 2i). Oil red O staining of aortic root sections and whole aorta also demonstrated reduced atherosclerotic lesions area in HMD‐fed *ApoE* ^−/−^ mice injected with Lv‐miR‐195‐3p (Figure 2j and Figure S1i), indicating miR‐195‐3p overexpression protects mice from the development of atherosclerosis. Importantly, the inhibition of inflammation was illustrated by ELISA and qRT‐RCR analysis, showing a significant decreased content and mRNA levels of IL‐31, IL‐1β, IL‐6 and TNF‐α both in serum and the aortic from HMD‐fed *ApoE* ^−/−^ mice injected with Lv‐miR‐195‐3p (Figure 2k), meaning miR‐195‐3p may exert an anti‐inflammation effect in HMD‐fed *ApoE* ^−/−^ mice. Taken together, these results demonstrated that miR‐195‐3p overexpression can ameliorate the atherosclerotic plaque formation in *ApoE* ^−/−^ mice by inhibiting macrophage inflammation through IL‐31.

### 2.3 Homocysteine facilitates DNA hypermethylation and H3K9 deacetylation of miR‐195‐3p promoter

**FIGURE 3:**
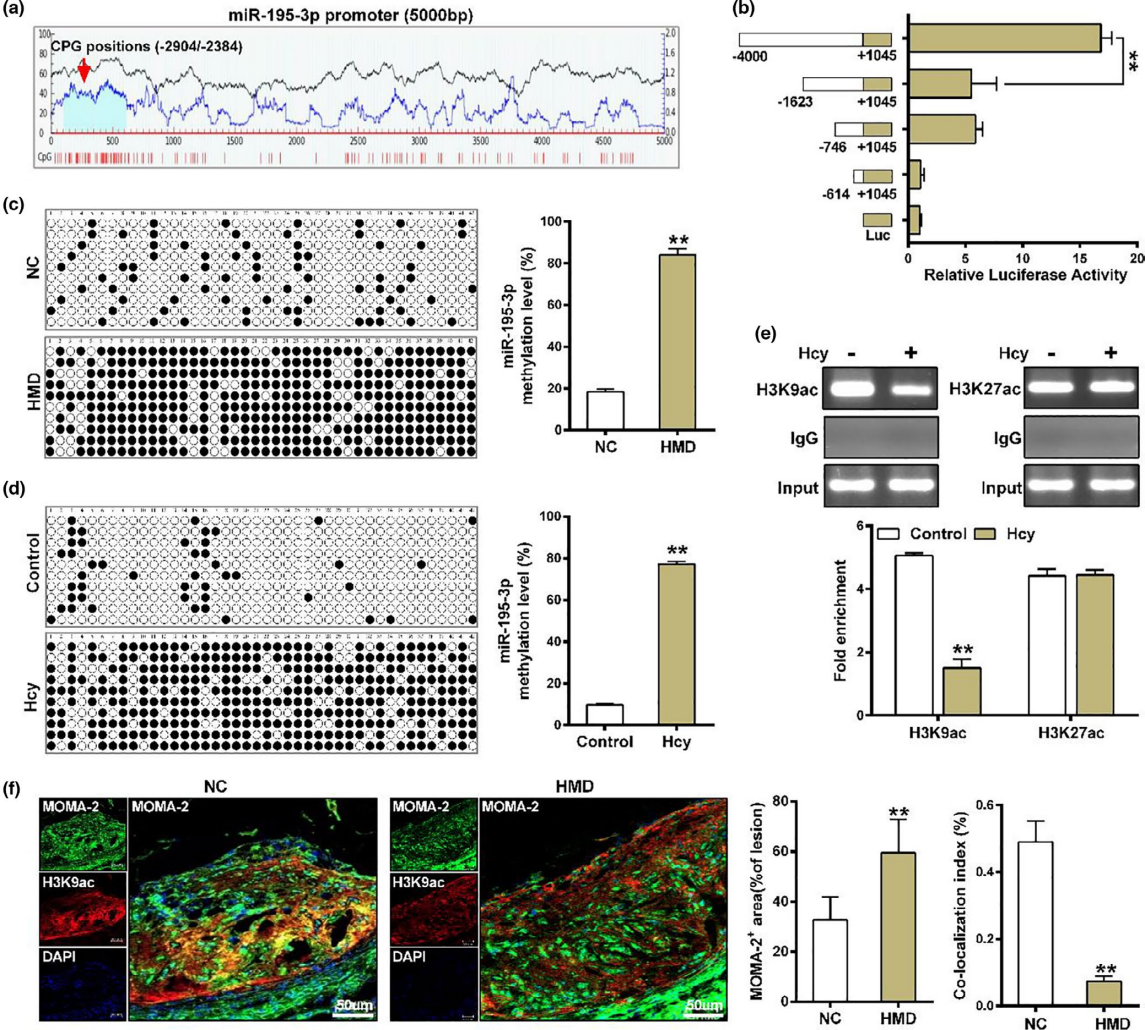
Homocysteine induces DNA hypermethylation and H3K9 deacetylation of miR‐195‐3p promoter in macrophages. (a) A schematic diagram of CpG islands in the miR‐195‐3p promoter by using the online accessible software MethPrimer. Vertical red lines denoted CpG sites, and CpG islands shown as light blue areas. (b) The promoter activity of miR‐195‐3p was evaluated by Dual‐Luciferase reporter assay. Different deletion fragments of the miR‐195‐3p promoter (−614/+1045, −746/+1045, −1623/+1045, and −4000/+1045) co‐transfected into HEK293 cells with renilla luciferase vector (internal control), and the results were represented as firefly luciferase activity normalized to renilla luciferase activity. (c, d) The methylation status of miR‐195‐3p promoter were evaluated by bisulfite sequencing PCR (BSP) in the aorta from HMD‐fed *ApoE* ^−/−^ mice and macrophages treated with Hcy. The cloned BSP was performed on the miR‐195‐3p promoter region (−2904 to −2384) relative to the transcription start site (TSS). The white cycle indicates unmethylated CpG dinucleotides whereas the black cycle represents methylated CpG dinucleotides. The percentage of methylation on each CpG dinucleotide was calculated by the number of methylated clones in each CpG site divided by the total number of clones in the same CpG site and shown in the right panel. (e) Chromatin immunoprecipitation (ChIP) assay of H3K9ac and H3K27ac occupancy at miR‐195‐3p promoter in macrophages treated with Hcy. Chromatin amplified with DNA fragment without antibody precipitation after sonication was used as Input, and IgG was amplified with DNA fragment precipitated by normal mouse IgG. The results were standardized with input DNA, and the data are the percentages of input chromatin. (f) Representative images and quantification of MOMA‐2 and H3K9ac levels by immunofluorescence staining in atherosclerotic plaque from *ApoE* ^−/−^ mice. Nuclei were stained with DAPI (blue). Scale bar =50 μm. Data were presented as mean ±SD. **p < 0.01

Transcriptional activity is the key to gene expression, which is susceptible to paradigmatic epigenetic marker, such as DNA methylation (Wang et al., 2020). We first analyzed the promoter sequence of miR‐195‐3p using MethPrimer (http://www.urogene.org/methprimer/), and a CpG island between −2,904 bp to −2,384 bp was observed in miR‐195‐3p promoter (Figure 3a). Meanwhile, several fragments (−4,000/+1,045, −1,623/+1,045, −746/+1,045, −614/+1,045) of miR‐195‐3p promoter were inserted into the firefly luciferase vector pGL3, and luciferase reporter assay showed that the fragment (−4,000/−1,623) had the highest transcriptional activity (Figure 3b), meaning the fragment (−4,000/−1,623) is the miR‐195‐3p basic core regulation region, which provides the structural basis for methylation modification. Therefore, miR‐195‐3p methylation status of 42 CpGs from −2,904 bp to −2,384 bp relative to the transcription start site (TSS) was characterized by bisulfite genomic sequencing. The results showed that miR‐195‐3p promoter has a general hypermethylation status in the aorta of HMD‐fed *ApoE* ^−/−^ mice (Figure 3c). Likewise, hypermethylation of miR‐195‐3p promoter was also observed in macrophages treated with Hcy (Figure 3d). These results indicated that Hcy‐induced hypermethylation of miR‐195‐3p promoter. In addition to DNA methylation, a possible mechanism to explain gene transcription regulation is histone acetylation (Martin et al., 2021). We next analyzed an association of the potentially chromatin modification patterns of miR‐195‐3p by using UCSC database (https://genome.ucsc.edu/) and found that H3K9ac and H3K27ac may occur at 5′ upstream regions of miR‐195‐3p (Figure S2). ChIP assay was applied to measure the enrichment of H3K9ac and H3K27ac at miR‐195‐3p promoter, and the results indicated that H3K9ac but not H3K27ac was significantly decreased at miR‐195‐3p promoter in macrophages treated with Hcy (Figure 3e). Consistent with the ChIP result, immunofluorescence staining also showed a significant decrease of H3K9ac predominantly co‐localized with MOMA‐2 positive cells in atherosclerotic plaque from HMD‐fed *ApoE* ^−/−^ mice (Figure 3f). These data suggested that Hcy‐induced DNA hypermethylation and H3K9 deacetylation of miR‐195‐3p promoter.

### 2.4 Homocysteine downregulation of miR‐195‐3p expression via synergy of DNMT3a and HDAC11

**FIGURE 4:**
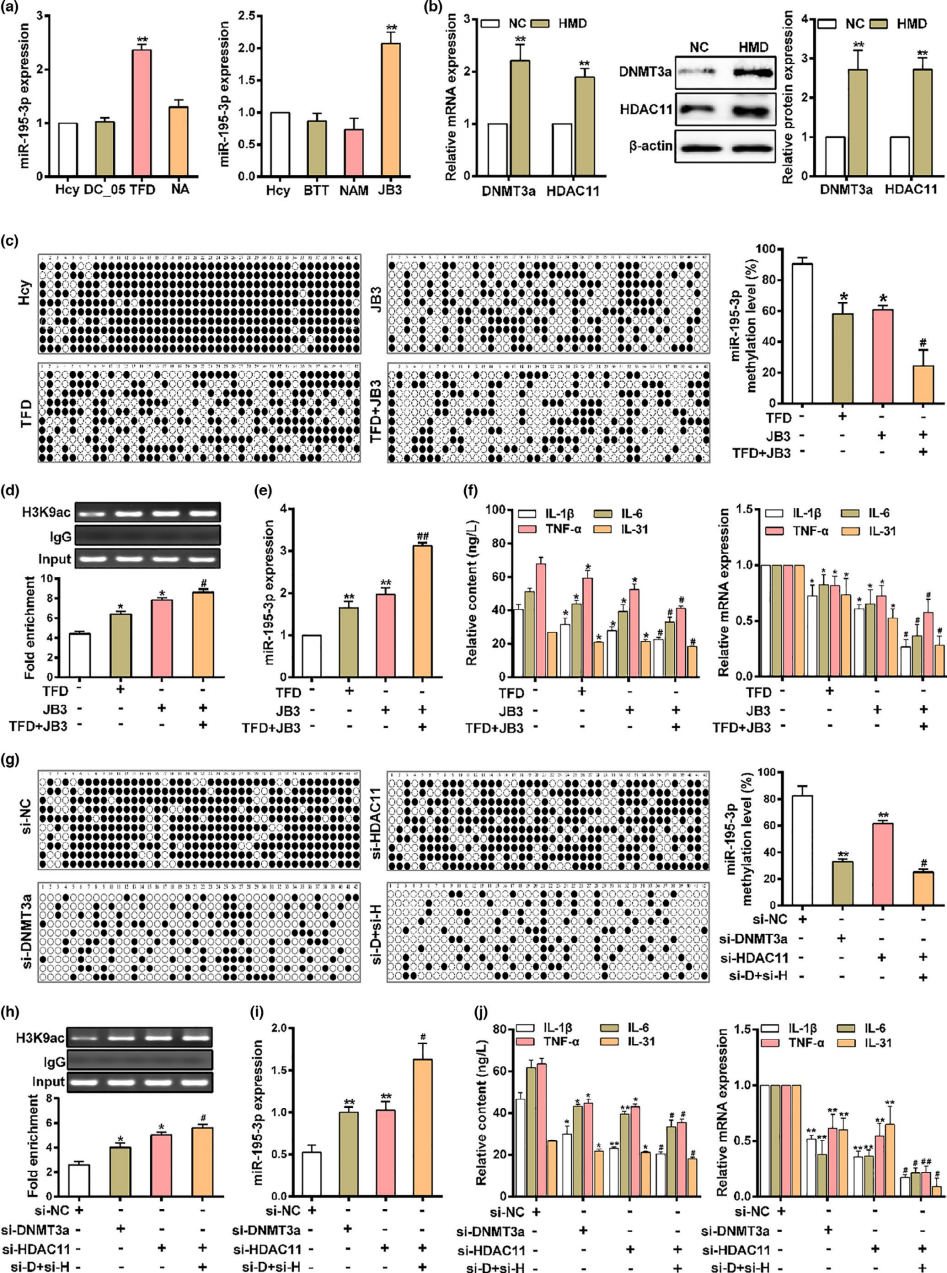
DNMT3a and HDAC11 play a pivotal role in the inhibition of miR‐195‐3p expression induced by Homocysteine. (a) The change of miR‐195‐3p in macrophages treated with DNMTs specific inhibitor or HDACs specific inhibitor in presence of Hcy. Among them, DC_05, Theaflavin‐3, 3′‐digallate (TFD) and Nanaomycin A (NA) are specific inhibitor of DNMT1, DNMT3a and DNMT3b, respectively. Butyrate (BTT), Nicotinamide (NAM) and JB3 are specific inhibitor of HDAC I/II, HDAC III and HDAC11, respectively. (b) The expression of DNMT3a and HDAC11 was measured in the aorta from *ApoE* ^−/−^ mice by qRT‐PCR and western blot, respectively. (c) DNA methylation status of miR‐195‐3p promoter was performed by BSP in macrophages treated with TFD and JB3 in presence of Hcy. (d) The H3K9ac occupancy at miR‐195‐3p promoter in macrophages treated with TFD and JB3 in presence of Hcy was determined by ChIP assay. (e) The expression of miR‐195‐3p in macrophages treated with TFD and JB3 in presence of Hcy were determined by qRT‐PCR. (f) The secretion and mRNA expression of IL‐1β, IL‐6, TNF‐α and IL‐31 were evaluated by ELISA and qRT‐PCR, respectively. (g) Methylation status of miR‐195‐3p promoter in macrophages transfected with siRNAs against DNMT3a (si‐DNMT3a) and HDAC11 (si‐HDAC11) in presence of Hcy was evaluated by BSP. (h) ChIP analysis of H3K9ac at miR‐195‐3p promoter by using anti‐H3K9ac in macrophages transfected with si‐DNMT3a and si‐HDAC11 in presence of Hcy. (i) qRT‐PCR was performed to determine the miR‐195‐3p expression in macrophages transfected with si‐DNMT3a and si‐HDAC11 in presence of Hcy. (j) ELISA and qRT‐PCR were used to determine the secretion and mRNA expressions of IL‐1β, IL‐6, TNF‐α and IL‐31 in macrophages transfected with si‐DNMT3a and si‐HDAC11 in presence of Hcy. Data were presented as mean ±SD. *p < 0.05, **p < 0.01; ^#^ *p* < 0.05, ^##^ *p* < 0.01

As the above data suggested that the DNA methylation and H3K9 deacetylation of miR‐195‐3p promoter support miR‐195‐3p transcription regulation, we next examined the key enzymes involved in Hcy‐mediated DNA methylation and histone acetylation. As shown in Figure 4a, macrophages treatment with theaflavin‐3, 3′‐digallate (TFD, DNMT3a specific inhibitor) or JB3 (HDAC11 specific inhibitor) increased the expression of miR‐195‐3p, meaning that DNMT3a and HDAC11 are involved in the regulation of miR‐195‐3p expression. Meanwhile, we observed significant increased DNMT3a and HDAC11 expression in HMD‐fed *ApoE* ^−/−^ mice (Figure 4b). Considering that DNA methylation could not exert its function in isolation within the context of chromatin, as there exists a complex interplay between DNA methylation and histone modifications, we speculated that DNA methylation and histone acetylation can interact with each other to regulate miR‐195‐3p transcription. As shown in Figure 4c,d, DNA methylation level and H3K9 deacetylation of miR‐195‐3p promoter were notably decreased in macrophages with treatment of TFD or JB3 alone in presence of Hcy, and a further decrease was observed when using them jointly, which accompanied by the opposite alteration of miR‐195‐3p expression (Figure 4e). This prompted us to study whether DNMT3a and HDAC11 could improve macrophage inflammation in atherosclerotic plaque formation. The macrophage inflammation was correspondingly promoted as evidenced by the marked increase of secretion and mRNA expression of IL‐1β, IL‐6, TNF‐α and IL‐31 (Figure 4f). Similar trends were found after macrophages transfected with siRNAs against DNMT3a (si‐DNMT3a) and siRNAs against HDAC11 (si‐HDAC11) (Figure S3a,b). The results showed that inhibition of DNMT3a and HDAC11 could attenuate the levels including DNA methylation, H3K9 deacetylation in miR‐195‐3p promoter and macrophage inflammation, while increased miR‐195‐3p expression (Figure 4g–j). In addition, a stronger effect was found in macrophages after treatment with si‐DNMT3a and si‐HDAC11 collectively. These results indicated that DNMT3a and HDAC11 at miR‐195‐3p promoter play a synergistic role in the suppression of miR‐195‐3p expression, thereby improve macrophage inflammation induced by Hcy.

### 2.5 Sp1 enhances miR‐195‐3p expression by direct binding to its promoter

**FIGURE 5:**
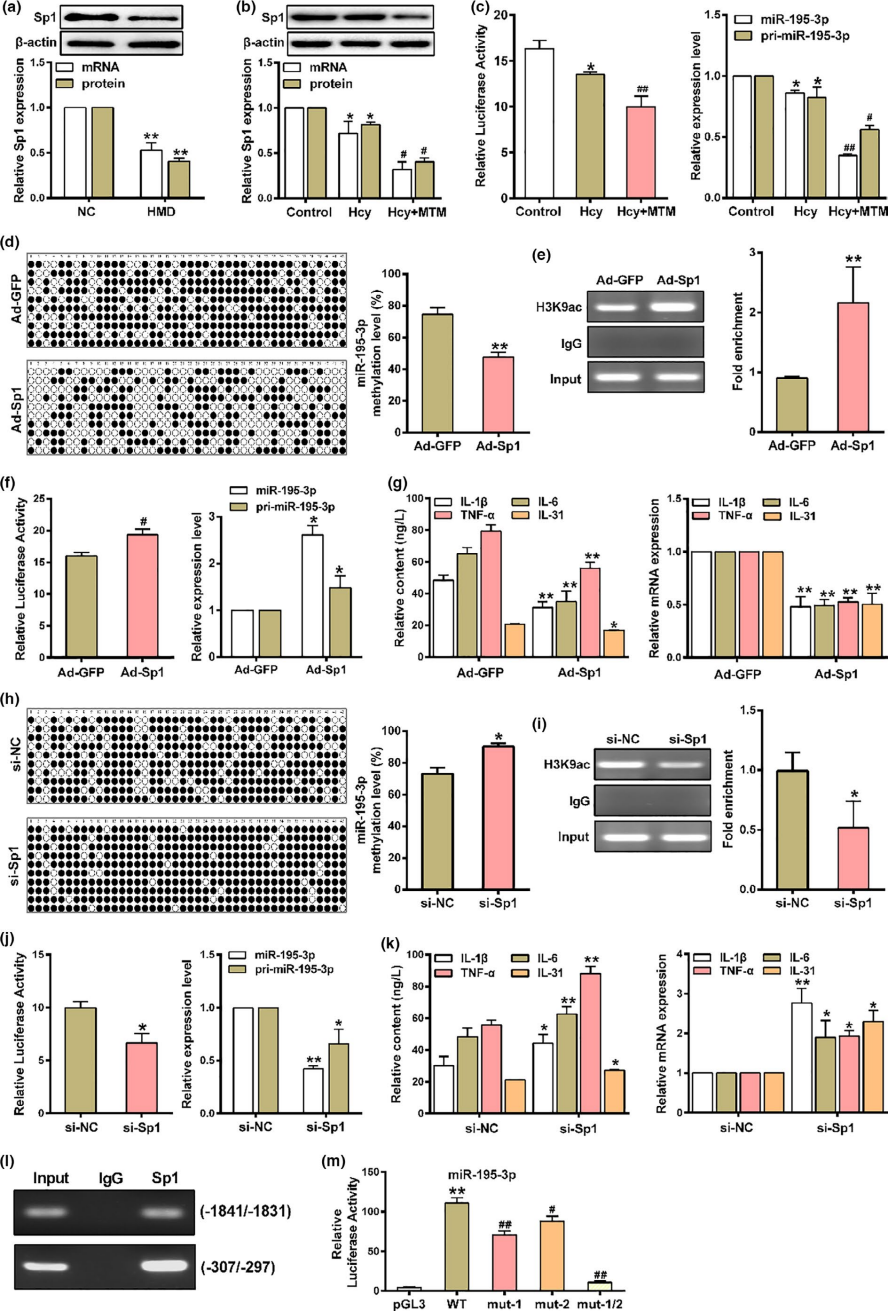
Sp1 increases the transcription of miR‐195‐3p by directly binding to its promoter. (a) The expression of Sp1 in the aorta of *ApoE* ^−/−^ mice were measured by qRT‐PCR and western blot. (b) qRT‐PCR and western blot determined the expression of Sp1 in macrophages treated with Hcy or/and mithramycin A (MTM, Sp1 specific inhibitor). (c) The promoter activity of miR‐195‐3p, the expression of miR‐195‐3p and pri‐miR‐195‐3p were measured by luciferase reporter assay and qRT‐PCR after cells treated with Hcy or/and MTM. (d) The levels of miR‐195‐3p methylation in promoter was analyzed by BSP, and (e) the levels of H3K9ac at miR‐195‐3p promoter were detected by ChIP, and (f) the promoter activity of miR‐195‐3p, the expression of miR‐195‐3p and pri‐miR‐195‐3p were measured by luciferase reporter assay and qRT‐PCR, and (g) the secretion and mRNA expression of IL‐1β, IL‐6, TNF‐α and IL‐31 were determined by ELISA and qRT‐PCR in macrophages transfected with the adenovirus expressing Sp1 (Ad‐Sp1) or Ad‐GFP in presence of Hcy. (h) The methylation status of the miR‐195‐3p promoter, and (i) H3K9ac occupancy at miR‐195‐3p promoter were detected by ChIP, and (j) the promoter activity of miR‐195‐3p, the expression of miR‐195‐3p and pri‐miR‐195‐3p were measured by luciferase reporter assay and qRT‐PCR, and (k) the IL‐1β, IL‐6, TNF‐α and IL‐31 secretion and mRNA expression were determined by ELISA and qRT‐PCR in macrophages transfected with siRNAs against Sp1 (si‐Sp1) or control siRNA (si‐NC) in presence of Hcy. (l) Sp1 binding at miR‐195‐3p promoter in macrophages was assessed by ChIP using an anti‐Sp1 antibody. The Sp1‐enriched miR‐195‐3p promoter containing the putative Sp1 binding sites was amplified by PCR. (m) Sequential deletion and substitution mutation analyses identified Sp1‐responsive regions in miR‐195‐3p proximal promoter region, the relative luciferase activities of miR‐195‐3p promoter in macrophages co‐transfected with Ad‐Sp1 and solely or serially truncated Sp1 binding sites at miR‐195‐3p promoter were detected using luciferase reporter assay. Data were presented as mean ±SD. *p < 0.05, **p < 0.01; ^#^ *p* < 0.05, ^##^ *p* < 0.01

Sp1, a prototypic C2H2‐type zinc finger protein, has been reported to activate or repress gene transcription in response to physiologic or pathologic stimuli (Vizcaíno et al., 2015). To examine whether Sp1 operates by a similar mechanism in macrophages during atherosclerotic plaque formation, we first analyzed miR‐195‐3p promoter sequence using the Searching Transcription Factor Binding Sites (TFsearch) (http://diyhpl.us/~bryan/irc/protocol‐online/protocol‐cache/TFSEARCH.html). This bioinformatic analysis revealed that two putative Sp1 binding sites (−1,841 to −1,831 and −307 to −297) were located at miR‐195‐3p promoter region (Figure S4a), suggesting regulation of miR‐195‐3p transcription may be correlated with Sp1. Next, we detected Sp1 expression in atherosclerotic plaque of *ApoE* ^−/−^ mice. The results showed that the expression of Sp1 were significantly decreased in HMD‐fed *ApoE* ^−/−^ mice compared with NC‐fed *ApoE* ^−/−^ mice (Figure 5a). Furthermore, Hcy led to a marked decrease Sp1 expression in macrophages, which were further reduced by Sp1 specific inhibitor mithramycin A (MTM) (Figure 5b). In addition, the promoter activity of miR‐195‐3p and the expression level of pri‐miR‐195‐3p as well as mature miR‐195‐3p were significantly decreased in macrophages treated with MTM (Figure 5c). These results suggested that Sp1 may be involved in the transcriptional regulation of miR‐195‐3p. To explore the interaction between miR‐195‐3p and Sp1, the adenovirus expressing Sp1 (Ad‐Sp1) and siRNAs against Sp1 (si‐Sp1) were transfected with macrophages (Figure S4b,c). Of note, macrophages transfected with Ad‐Sp1 significantly reduced DNA methylation of miR‐195‐3p promoter, while H3K9ac of miR‐195‐3p promoter was elevated (Figure 5d,e). It was further found that overexpression of Sp1 promote miR‐195‐3p transcriptional activity and the expression of pri‐miR‐195‐3p as well as mature miR‐195‐3p, which was accompanied by the decreased secretion of pro‐inflammatory factors (Figure 5f,g). Conversely, silencing Sp1 obtained the opposite results (Figure 5h–k), suggesting that Sp1 positively regulate miR‐195‐3p in macrophages inflammation induced by Hcy. To further assess that the binding of Sp1 to the predicted sites in miR‐195‐3p promoter, ChIP assay was performed with anti‐Sp1 antibody. As shown in Figure 5l, Sp1 bound to the miR‐195‐3p promoter at −1,841 to −1,831 and −307 to −297 sites. More importantly, by combining substitution mutations of the sites and luciferase reporter assay, we found that either of the two binding sites mutations could result in a decreased transcriptional activity of miR‐195‐3p, and a further reduction was observed when mutation of the two binding sites (Figure 5m), suggesting the importance of these two Sp1 binding sites in miR‐195‐3p transcriptional activation. Collectively, these results demonstrated the positive regulation of Sp1 on miR‐195‐3p expression in atherosclerosis.

### 2.6 Sp1 interacts with DNMT3a to suppress HDAC11 binding to miR‐195‐3p promoter

**FIGURE 6:**
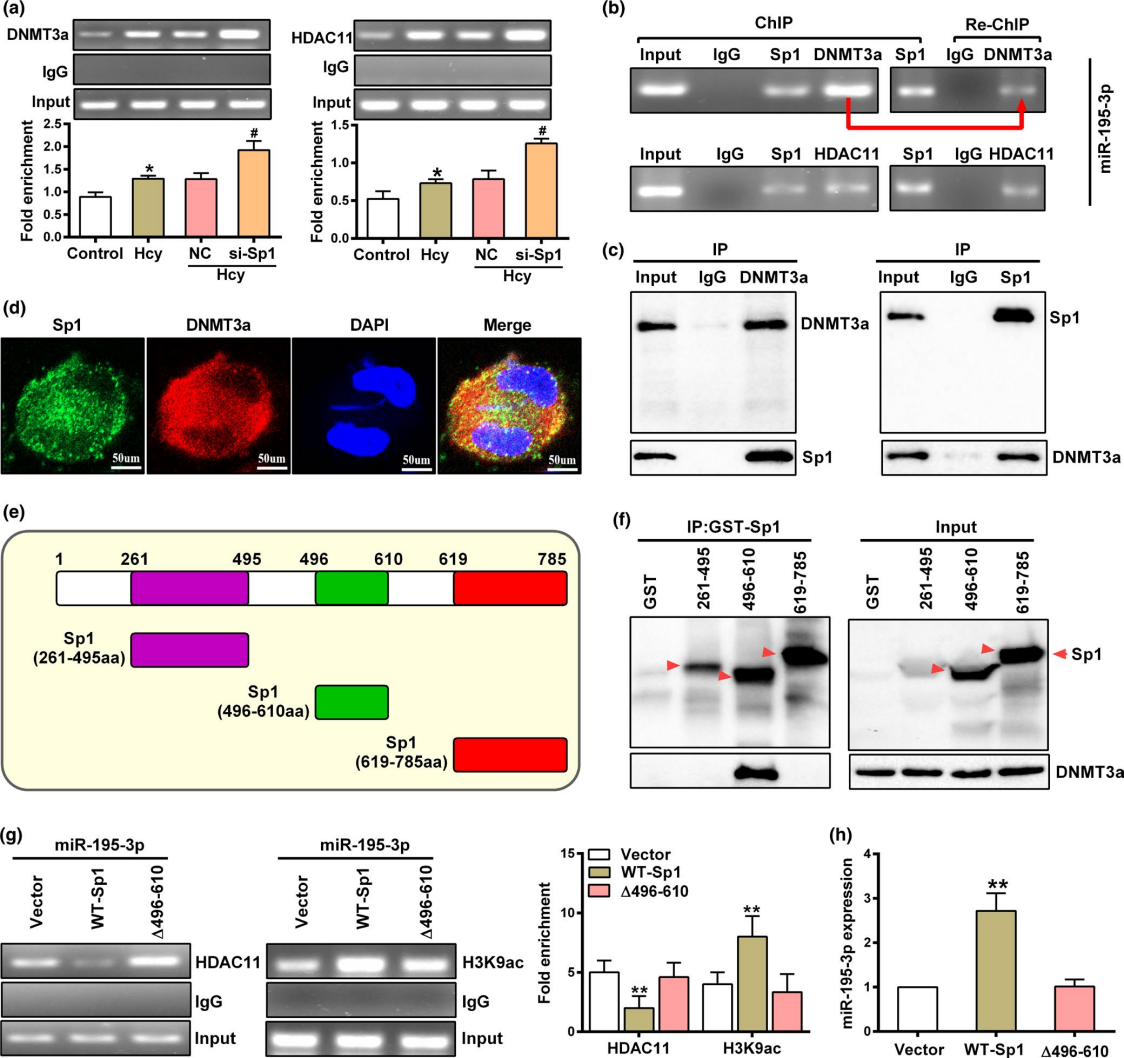
Sp1 inhibits HDAC11 binding to miR‐195‐3p promoter by binding to DNMT3a. (a) The enrichment of DNMT3a or HDAC11 at miR‐195‐3p promoter were determined by ChIP in macrophages after transfected with si‐NC or si‐Sp1. (b) ChIP analysis with anti‐Sp1 antibody was eluted and subjected to a Re‐ChIP using either the anti‐DNMT3a or HDAC11 antibody in macrophages treated with Hcy. Representative gels shown the PCR product performed with primers flanking the Sp1 binding regions at miR‐195‐3p promoter. (c) The interaction between Sp1 and DNMT3a was analyzed by Co‐Immunoprecipitation (Co‐IP) in macrophages treated with Hcy. (d) Immunofluorescent images shown the co‐localization of Sp1 (green) and DNMT3a (red) in Hcy‐treated macrophages using laser confocal microscopy, and nuclei were stained with DAPI (blue). Scale bar =50 μm. (e) Schematic diagram depicting the structure of Sp1 and truncation mutants of the GST‐tagged Sp1 subunit. (f) GST pull‐down assays were performed using recombinant GST, GST‐(261–495), GST‐(496–610) and GST‐(619–785) to pull‐down DNMT3a. Bound and 10% of input DNMT3a were detected by western blot using an anti‐HA antibody. Specific GST‐Sp1 mutants are indicated by red arrows. (g) The enrichment of HDAC11 and H3K9ac at miR‐195‐3p promoter in Hcy‐treated macrophages infected with wild‐type (WT‐Sp1) and mutant (Δ496‐610) was assessed by ChIP assay using an anti‐HDAC11 and H3K9ac antibody. (h) The expression of miR‐195‐3p in Hcy‐treated macrophages infected with wild‐type (WT) and mutant (Δ496‐610) adenoviral vector expressed Sp1 using qRT‐PCR. Data were presented as mean ±SD. *p < 0.05, **p < 0.01; ^#^ *p* < 0.05

Given that transcription activation of miR‐195‐3p is dependent on Sp1, we proceeded to explore the effect of Sp1 on the binding ability of DNMT3a and HDAC11 to miR‐195‐3p promoter. ChIP assay showed that silencing Sp1 in macrophages resulted in a significant increase binding of DNMT3a and HDAC11 to the miR‐195‐3p promoter after Hcy treatment (Figure 6a). Then, ChIP and Re‐ChIP assays were applied to explore the potential link between Sp1, DNMT3a, and HDAC11 in macrophages. ChIP assay shown that both Sp1, DNMT3a and HDAC11 bound to the miR‐195‐3p promoter after Hcy treatment (Figure 6b, left). Re‐ChIP assay further indicated that the enrichment of DNMT3a at miR‐195‐3p promoter was decreased, whereas no obvious change was observed for HDAC11 enrichment, meaning a close relationship between DNMT3a and Sp1 (Figure 6b, right). This prompted us to further examine the relationship between DNMT3a and Sp1 using Co‐IP analyses. As shown in Figure 6c, DNMT3a could be co‐immunoprecipitated with Sp1. Meanwhile, immunofluorescence staining confirmed the co‐localization of Sp1 and DNMT3a as many yellow spots was observed in macrophages treated with Hcy (Figure 6d). The consistent patterns suggesting the potential of the interaction between DNMT3a and Sp1. Subsequently, several functional domains of Sp1 were constructed, including two transactivation domains (261–495, 496–610) and VZV IE62‐binding (619–785) domain (Figure 6e). We firstly performed pull‐down assay using recombinant GST‐(261–495), GST‐(496–610) and GST‐(619–785) vectors, and the results exhibited that the regions spanning amino acids 496–610 confer strong interaction with DNMT3a (Figure 6f). We next constructed the mutants of Sp1, the recombinant which delete the sequence from 496 to 610 amino acids and performed the ChIP assay. Intriguingly, the results revealed a significant increased HDAC11 recruitment to miR‐195‐3p promoter with the mutation of Sp1 and DNMT3a binding site, which decreased H3K9ac of miR‐195‐3p promoter and its expression (Figure 6g,h), indicating the key role of structural domain for Sp1 to bind to DNMT3a. Collectively, these data strongly suggested that the amino acids from 496 to 610 of Sp1 was important for the interaction between DNMT3a and Sp1 to suppress HDAC11 at miR‐195‐3p promoter thus facilitate miR‐195‐3p transcription in macrophages.

## 3 DISCUSSION

The development of atherosclerosis and the incidence of its risk factors and clinical manifestations increase dramatically with age and are responsible for most cardiovascular morbidity and mortality in the elderly. The concept that inflammation has deleterious and additive effects in the onset and the progression of atherosclerosis and its complications has gained considerable attention (Gisterå & Hansson, 2017). Over the past decade, Hcy‐mediated pro‐inflammatory cytokines production and lipid accumulation in macrophages have been reported to promote the progression of atherosclerotic plaque (McCully, 2015a). Despite a wealthy of evidence indicated the potential benefits of treatment of inflammation in atherosclerosis, it remains a great threat to human health worldwide. The present study was aimed at investigating the effect of miR‐195‐3p on the inflammation of macrophages and atherosclerosis, and to elucidate the mechanisms by which miR‐195‐3p downregulation mediated by Hcy was regulated by both epigenetic modification and transcription mechanism. Collectively, these findings will provide a perspective on therapeutic opportunities for miRNAs in hyperhomocysteinemia (HHcy)‐associated cardiovascular diseases.

Accumulating evidences demonstrated that inflammation could drive the formation, progression, and rupture of atherosclerotic plaques (Gencer et al., 2021). Macrophages are an important component of vessel wall infiltrates, which exert the pathogenic effects on vascular inflammation mainly through the secretion of soluble factors, such as cytokines, growth factors and enzymes (Koelwyn et al., 2018). Hcy is a sulfur‐containing amino acid, and the elevated level of Hcy is considered toxic as the excess of which may be exported out from cells into systemic circulation (Azzini et al., 2020). Macrophages polarize into M1 and M2 distinct functional subpopulations exposed to environmental signals like Hcy (Winchester et al., 2015). Notably, M1 macrophages contribute to Th1 responses, and mediate inflammatory and tissue disruptive reactions through an armamentarium of pro‐inflammatory cytokines, including IL‐1β, IL‐6 and TNF‐α (Rigoni et al., 2020). The uncontrolled inflammation could hamper macrophages cholesterol efflux and efferocytosis directly, which result in the accumulation of debris and enlargement of necrotic cores in atherosclerotic plaque (Baardman et al., 2020). Moreover, inflammatory mediators released by plaque macrophages aggravate local tissue damage, which in turn arouses more inflammation and forms a vicious circle (Deng et al., 2020). In this study, our results showed that Hcy promotes the production of pro‐inflammatory cytokines IL‐1β, IL‐6 and TNF‐α in macrophages during the formation of atherosclerotic plaques, suggesting that macrophage inflammation might be a critical target of the atherogenic effect of Hcy.

It is widely accepted that epigenetic and genetic alterations are responsible for the initiation and progression of atherosclerosis (Xu et al., 2018). In recent years, miRNAs as one of an epigenetics modifications have attracted attention for its role in atherosclerosis. In addition, it has been reported that miRNAs modulate gene expression at the post‐transcriptional level either by inhibiting messenger RNA (mRNA) translation or by promoting mRNA degradation (Michlewski & Cáceres, 2019). Previous studies have reported that miR‐126 could inhibit the production of inflammatory cytokines through MAPK signaling pathway to prevent atherosclerosis progression and development (Hao & Fan, 2017). miR‐195, a member of the miR‐15 family, was often downregulated in various types of cancers, such as breast cancer and non‐small cell lung cancer (Luo et al., 2019; Yang et al., 2019). Interestingly, miR‐195‐5p alleviates acute kidney injury through repression of inflammation and oxidative stress by targeting VEGFA (Xu et al., 2020). Our study found that elevated Hcy downregulated miR‐195‐3p expression to facilitate the macrophage inflammation through targeting IL‐31. Importantly, injection of Lv‐miR‐195‐3p into *ApoE* ^−/−^ mice could alleviate the pathological process of atherosclerosis through the inhibition of pro‐inflammatory cytokines production. This observation suggests that miR‐195‐3p might be a candidate for Hcy‐induced macrophage inflammation and atherosclerotic plaque formation. However, more investigations are needed to fully uncover the detailed underlying mechanism.

Similar to protein‐coding genes, miRNAs expression is also controlled by epigenetic modifications, including DNA methylation, histone modifications, and chromatin remodeling, which are susceptible to the environment factors (Yao et al., 2019). DNA methylation is one of the most well characterized epigenetic phenomena, which is typically mediated by DNA methyltransferases (DNMTs, including DNMT1, DNMT3a and DNMT3b) and requires the presence of S‐adenosylmethionine (SAM), a methyl donating compound that delivers methyl groups to maintain other metabolic reactions in mammals (Horvath & Raj, 2018). Hcy is an intermediate product released during methionine cycle. Methionine combines with adenosine to produce SAM, which is converted to SAH and further hydrolyzed to Hcy and adenosine after releasing its methyl group, suggesting the critical importance for Hcy‐methionine cycle in the maintenance of a balanced amount of SAM, and normal DNMTs activity (Greco et al., 2020). We sought to understand the miR‐195‐3p regulation mechanism in Hcy‐induced atherosclerosis, we found that the promoter of miR‐195‐3p contains one CpG island and 42 CpG sites. Moreover, miR‐195‐3p promoter DNA hypermethylation has been detected due to the increased binding of DNMT3a at its promoter after Hcy treatment. This point was supported by our previous study that Hcy‐induced DNA hypermethylation could trigger autophagy by suppressing CFTR expression in the liver of *ApoE* ^−/−^ mice (Yang et al., 2018).

Interestingly, Hcy has been reported to induce differential levels of DNA methylation, range from hyper‐ to hypomethylation, which could be explained by other mechanisms in regulating DNA methylation (Mandaviya et al., 2014). Recently, it has been shown that DNMT1 recruited HDAC1 to repress gene expression or to form heterochromatin structures (Ma et al., 2021). Some studies supported that DNA methylation can provide binding sites for methyl‐binding domain proteins (MBDs), which can interact with HDACs to mediate gene repression (Pandey et al., 2014). Besides, Hcy can induce H3K9ac and inhibit the expression of HDAC1 in foam cells (Zhao et al., 2017). In agreement with these observations, our results found that Hcy induces H3K9 deacetylation and HDAC11 binding at miR‐195‐3p promoter, indicated cooperative interaction between DNA methylation and histone acetylation in the mediation of transcriptional repression. Nevertheless, although the DNA methylation and H3K9ac were found to be involved in the reduction of miR‐195‐3p in Hcy‐mediated atherosclerosis, we cannot completely exclude the possibility that the effects of Hcy on other mechanisms to alter miR‐195‐3p expression during atherosclerosis. Therefore, more delicate investigations are needed for a complete understanding of the mechanisms of miR‐195‐3p in Hcy‐mediated atherosclerosis.

Gene expression program is largely regulated by the inducible expression of transcription factors in response to the specific stimuli. DNA methylation and histone acetylation occurring in a regulatory region could work together to alter the binding profile of transcription factors and the specific gene expression (Cusack et al., 2020). Sp1 is one of the transcription factors that regulate the inflammation‐repair process. Previous study reported that modulation of NF‐κB and Sp1 expression may be important targets for the prevention and treatment of atherosclerotic vascular disease (Lee et al., 2013). In this study, we found two putative Sp1 binding sites at miR‐195‐3p promoter, which could recruit DNMT3a and HDAC11. Meanwhile, our work further demonstrates that Sp1 suppress macrophage inflammation which contribute to the overall decrease in plaque formation.

Since Hcy is involved in the methionine cycle by using as a methyl group carrier, in which the methyl group of Hcy is transferred to not only DNA but also histones and other proteins, the presence of methyl group at a specific CpG dinucleotide site may directly prevent DNA from recognizing and binding to transcription factors (Lai et al., 2020). Furthermore, methylated DNA can prevent the binding of HDACs, which can catalyze histone modification and form chromatin‐remodeling complexes, leading to altered structure of chromatin and transcription repression (Pasyukova et al., 2021). Epigenetic regulation on transcription is initiated by specific transcription factors that bind to the promoters of genes upon altering transcription. These factors promote changes in chromatin structure and binding of RNA polymerase II (RNAPII) to promote future transcriptional reactivation (Álvarez‐Errico et al., 2015; Le et al., 2020). These findings correlate with DNA methylation and chromatin changes, this prompted us to study the specific mechanism of Sp1 regulating miR‐195‐3p transcription. The present study further uncovered that the binding region between Sp1 and DNMT3a are necessary for HDAC11 binding to the miR195‐3p promoter. Thus, these results indicated that the therapeutic promises of miR‐195‐3p in treating Hcy‐induced atherosclerosis and clarified the importance of Sp1 in the regulation of DNA methylation and H3K9ac to be involved in Hcy‐mediated miR‐195‐3p downregulation.

In summary, our results suggested that Hcy‐mediated miR‐195‐3p promotes macrophage inflammation and the progress of atherosclerosis by targeting IL‐31. More importantly, DNA methylation and H3K9 deacetylation catalyzed by DNMT3a and HDAC11 are responsible for the downregulation of miR‐195‐3p, and Sp1 interacts with DNMT3a was essential for HDAC11 to bind to miR‐195‐3p promoter. Given the reversibility of epigenetic changes, our study aimed to restore miR‐195‐3p expression to be beneficial for cardiovascular patients, particularly for HHcy‐related atherosclerosis.

## 4 EXPERIMENTAL PROCEDURES

### 4.1 Animal treatment

6‐week‐old apolipoprotein E knockout (*ApoE* ^−/−^) male mice purchased from the Laboratory Animal Center of Peking University Health Science Center (Beijing, China) were housed individually at room temperature, provided food and water ad libitum. After 2 weeks adaption, they were randomly divided into NC group (regular diet: 20% protein, 4.5% fat, 55.5% carbohydrate) and HMD group (high methionine diet: 1.7% methionine). Frozen sections of aortic root and the entire aorta were stained with Hematoxylin‐eosin (HE) or Oil red O for quantification of the lesion area. Aortic lesion size of each animal was obtained by averaging the lesion areas in three sections from the same mice. Boundary lines were drawn around these regions, and the area measurements were obtained by ImageJ software. Recombinant lentiviruses expressing miR‐195‐3p (Lv‐miR‐195‐3p) or Lv‐miR‐neg were injected to HMD fed‐*ApoE* ^−/−^ mice via tail vein, and once every 4 weeks. The titer of the virus was 2 × 10^9^ TU/ml, and an equal volume of 50 μl phosphate buffered solution (PBS) was used as vehicle control. After 20th weeks, mice were killed and blood was collected for analysis of Hcy, total cholesterol (TC), triglyceride (TG), free cholesterol (FC), IL‐31, IL‐1β, IL‐6 and TNF‐α levels. All animals received humane care in compliance with the Institutional Authority for Laboratory Animal Care of Ningxia Medical University following the Guide for the Care and Use of Laboratory Animals published by the United States National Institutes of Health.

### 4.2 Ultrasound biomicroscopy (UBM)

The mice were anesthetized and laid supine. The warm ultrasonic transmission gel was placed on the chest. The mechanical transducer (Vevo 770, Visual Sonics, Toronto, Canada) was set at 40 MHz, and the Real‐Time Micro Visualization Scanhead (RMV 704) was used for intima‐media thickness (IMT) and blood velocity measuring. All mice were measured at least 3 times, and the measurement data were statistically analyzed.

### 4.3 Immunofluorescence

Frozen aortic roots sections or macrophages were fixed in 4% paraformaldehyde for 15 min and permeabilized in 0.2% Triton X‐100 for 15 min. After blocking with 10% goat serum, the tissue sections or macrophages were indicated with primary antibodies at 4°C overnight. After washing with PBS for 3 times, tissue sections or macrophages were incubated with fluorescein‐conjugated secondary antibodies (Santa Cruz Biotechnology) for 2 h at room temperature. The cell nuclei were stained by incubation with 4, 6‐diamidino‐2‐phenylindole (DAPI, Bioss, Woburn, MA, USA). The fluorescence was detected and photographed by confocal microscopy (Olympus, Tokyo, Japan). The fluorescence intensity was quantified with ImageJ software, and relative fluorescence intensity was calculated as the targeted fluorescence intensity relative to the DAPI fluorescence intensity. Besides, the co‐localization analysis was performed using co‐localization plug‐in for ImageJ after converted the individual fluorescent channel images to 8‐bit grayscale.

### 4.4 In situ hybridization

For the detection of miR‐195‐3p in atherosclerotic plaques, frozen aortic roots sections were subjected to in situ hybridization. In brief, denature FAM‐labeled miR‐195‐3p LNA™ probe (Exiqon, Denmark) at 90°C and dilute the probe in Exiqon ISH buffer. Then tissue sections were treated with the slides with freshly prepared acetylation buffer for 7 min and washed 3 times in RNAse‐free PBS at room temperature. Remove PBS and immediately add 50 μl probe solution to slides and start a preset hybridization program for 1 h at 60°C. Following wash slides 3 times for 10 min using 55°C prewarmed 0.1× SSC, the slides were incubated with 200 μl blocking solution for 15 min at room temperature. Next, after incubated with anti‐FAM for 60 min, the slides were then incubated 150 μl freshly prepared NBT‐BCIP reagent for 2 h. Finally, wash each slide with KTBT buffer for 3 min and immunofluorescence staining with anti‐MOMA‐2. The number of miR‐195‐3p positive macrophages as assessed by MOMA‐2 staining on serial sections and expressed as percentage of MOMA‐2^+^ cells positive for miR‐195‐3p.

### 4.5 miRNAs microarray

Total RNA from aorta of *ApoE* ^−/−^ mice were isolated using TRIzol Reagent (Invitrogen, Eugene, OR, USA) and the RNA integrity was examined by electrophoresis. Following labeled with Hy3 by using the miRCURY array labeling kit (Agilent Technologies, Santa Clara, CA, USA) and hybridization to a miRCURY LNA microRNA array (Agilent Technologies, Santa Clara, CA, USA). Microarray images were acquired by the Agilent G2565CA scanner (Agilent Technologies, Santa Clara, CA, USA) and analyzed by Agilent Feature Extraction Software (version 10.7). The differentially expressed miRNAs were screened out with |Log_2_Fold Change|≥2 and *p* < 0.05 as the thresholds. TargetScan, miRWalk and miRbase were applied to predict the target genes of miRNAs, respectively. Common genes predicted different algorithms were retained as target genes.

### 4.6 Cell culture and treatment

Macrophages were induced from human monocyte leukemia cell line (THP‐1) as described previously (Guo et al., 2020). Lv‐miR‐195‐3p, Lv‐miR‐neg, adenovirus encoding IL‐31 (Ad‐IL‐31), adenovirus expressing Sp1 (Ad‐Sp1), Ad‐GFP, siRNAs against Sp1 (si‐Sp1), siRNAs against DNMT3a (si‐DNMT3a), siRNAs against HDAC11 (si‐HDAC11) or control siRNA (si‐NC) were obtained from Genepharma (Shanghai, China) and infected as previously described (Guo et al., 2020). And they were transiently transfected into the cells using Lipofectamine 2000 (Life Technologies, Gaithersburg, MD, USA) following the manufacturer's instruction. The transfection efficiency was detected by qRT‐PCR and western blot, then the cells were collected for downstream analysis.

### 4.7 Quantitative real‐time polymerase chain reaction (qRT‐PCR)

Total RNA was extracted from aorta tissue or macrophages and then reverse‐transcribed into cDNA by using the Reverse Transcription Kit (Takara, Dalian, China). qRT‐PCR analysis was performed using the FTC3000 qRT‐PCR detection system (Funglyn Biotech Inc, Canada), and all reactions were performed thrice. Bulge‐Loop™ miRNA primers for miR‐195‐3p (RIBOBIO, Guangzhou, China) were used according to the manufacturer's protocols. The primers were listed in Table 1. Furthermore, the mRNA and miRNA expression were normalized using GAPDH or U6 as reference gene, and the relative gene expression was calculated using the 2^−ΔΔCt^ method.

**TABLE 1:** Primer sequences for qRT‐PCR analyses

Gene	Species	Genbank	Primer sequence, 5′ to 3′
GAPDH	Human	NM_002046.6	F: GCTCTCTGCTCCTCCTGTTC R: TTCCCGTTCTCAGCCTTGAC
IL−31	Human	NM_001014336.2	F: CGTCCGTTTACTACGACCAAGTG R: GGCAGCGTGTAATTCTGGGACA
IL−1β	Human	NM_000576.2	F: GTGGCAATGAGGATGACTTG R: TGGTGGTCGGAGATTCGTA
IL−6	Human	NM_000600.4	F: GGTGAGTGGCTGTCTGTGTG R: TTCGGTCCAGTTGCCTTCT
TNF‐α	Human	NM_000594.3	F: AGCCCATGTTGTAGCAAACC R: GCTGGTTATCTCTCAGCTCCA
DNMT3a	Human	NM_175629.2	F: GAGAACTGCAGGGCGAAGG R: TGTTGAGCCCTCTGGTGAAC
HDAC11	Human	NM_024827.3	F: GCTCTGCCCCCAGAGGA R: CTCTGGCACATGCTGGTACA

Abbreviations: F, forward; R, revers.

### 4.8 Western blot

Macrophages and aorta tissue were lysed in cold lysis buffer. The proteins were separated by 10% sodium dodecyl sulfate polyacrylamide gel electrophoresis (SDS‐PAGE) and then transferred to PVDF membranes (Millipore, Billerica, MA, USA). After blocked with 5% non‐fat milk, the membranes were incubated with indicated primary antibodies at 4°C overnight. After 3 times washing, the membranes were incubated with horseradish peroxidase (HRP)‐lighted secondary antibodies for 1 h, and the protein expression was then detected by using the chemiluminescence kit (KeyGEN, Nanjing, China). Optical density of bands was quantified using densitometry and normalized to a β‐actin loading control.

### 4.9 Bisulfite sequencing PCR (BSP)

Genomic DNA was purified from macrophages and tissues using a QIAamp DNA Mini Kits (Qiagen, Hilden, Germany). 2 mg genomic DNA was converted with sodium bisulfite as previously described. The bisulfite‐treated miR‐195‐3p promoter containing 42 CpG dinucleotides was amplified with the primers 5′‐TTATAAAAAGGGGGGTTTTGGT‐3′ (sense) and 5′‐ACCRCCCAAACCAAATAAATC‐3′ (antisense). Then, the amplified PCR products were purified, and subcloned into the pGEMT‐Easy vector (Promega, Madison, WI). At least 10 clones were selected for sequencing and their methylation status was analyzed using web‐based analysis software QUMA (http://quma.cdb.riken.jp/). The percentage of methylated CpG dinucleotides was calculated to evaluate the methylation level at miR‐195‐3p promoter.

### 4.10 Luciferase reporter assay

Dual‐luciferase reporter assay was used to evaluate the activity of miR‐195‐3p promoter and the direct downstream target IL‐31. For promoter activity assay, the different fragments of miR‐195‐3p promoter were inserted into the pGL3‐ control plasmid, and the wild‐type (WT) and mutant‐type (Mut) 3′‐UTR of IL‐31 were cloned and inserted into pGL3‐control vector, respectively. Then, the HEK293T cells were plated in 24‐well plates and co‐transfected with above reporter constructs, renilla luciferase expression vector, an internal control (pGL3‐basic) or miR‐195‐3p mimics. Luciferase activities were measured by using the dual‐luciferase reporter assay system (Promega, USA) 48 h after transfection according to the manufacturer's protocol. Normalized firefly luciferase activity was compared between groups.

### 4.11 Chromatin immunoprecipitation (ChIP) and Re‐ChIP assay

Sp1 binding and H3K9ac levels at miR‐195‐3p promoter were examined by chromatin immunoprecipitation (ChIP) using the EZ‐ChIP kit according to the manufacturer's instruction (Millipore, Billerica, MA, USA). Briefly, cell sediments were crosslinked with 1% formaldehyde for 15 min. Crosslinked chromatin was sonicated to generate chromatin samples with an average fragment size of 200–1,000 bp. After precleaning with protein, A‐sepharose beads, samples were incubated with anti‐H3K9ac antibody (Abcam, Cambridge, MA, USA), anti‐Sp1 (Invitrogen, Carlsbad, CA, USA) or normal control IgG at 4°C overnight. Antibody‐chromatin complexes were then pulled down with protein A‐sepharose beads that had been blocked with BSA and salmon sperm DNA. After washing, the immunoprecipitated DNA was eluted and resolved electrophoretically on a 2% agarose gel. In Re‐ChIP assays, the complexes were eluted from the beads by incubation with 10 mM DTT at 37°C for 30 min after washing the protein‐G‐Sepharose beads from the primary IP, with a 50‐fold dilution with 1× sonication buffer, the eluates were subjected to IP with the second antibody. Subsequently, the immunoprecipitated DNA was subjected to PCR analyses by using primers targeting the promoter regions of miR‐195‐3p.

### 4.12 Co‐Immunoprecipitation (Co‐IP)

Cells washed for 3 times with PBS were lysed in cold lysis buffer followed by centrifugation at 12,000g for 15 min. The cell lysates were incubated with indicated antibodies for 1 h and then incubated with protein G‐Dyna beads (Thermo Fisher, 10003D) for 30 min at 4°C. The agarose beads were washed 3 times with cold lysis buffer, followed by elution with 40 μl of protein lysis buffer. Then, 10 μl of 5× loading buffer (Beyotime, Shanghai, China) was added, and the mixture was boiled for 5 min and subjected to western blot.

### 4.13 Pull‐down assay

Recombinant proteins contain GST, GST‐(261–495), GST‐(496–610) and GST‐(619–785) were produced in Escherichia coli, prior immobilization on glutathione‐sepharose, followed by incubation with 100 ng of recombinant DNMT3a (GenePharma, Shanghai, China). Interactions were evaluated by immunoblotting anti‐DNMT3a (Abcam, Cambridge, MA, USA) or anti‐GST (Abbkine, CA, USA).

### 4.14 Statistical analysis

GraphPad Prism 6.0 software was used for statistical analysis. All data are presented as Mean ±SD from at least three independent experiments. Differences between groups were analyzed by one‐way analysis of variance combined with the student's‐Newman‐Keuls post hoc test or the nonparametric Mann‐Whitney *U* test. Pearson test analyzed the correlation between serum Hcy levels and atherosclerotic lesion area. The values of *p* < 0.05 was considered as statistically significant.

## CONFLICT OF INTEREST

The authors declare that they have no conflicts of interest.

## AUTHOR CONTRIBUTION

Jiantuan Xiong and Fang Ma designed and performed experiments, analyzed data, and wrote the paper; Fang Ma, Ning Ding, Lingbo Xu performed experiments and analyzed data; Shengchao Ma and Anning Yang analyzed the RNA‐seq data; Yinju Hao performed some bioinformatics analysis; Huiping Zhang and Yideng Jiang initiated the study, organized, designed, and wrote the paper. All authors reviewed and approved the final submitted manuscript.

## Supporting information

App S1

## Data Availability

The data that support the findings of this study are available from the corresponding author upon reasonable request.
