# Supplementary material for: miR‐195‐3p alleviates homocysteine‐mediated atherosclerosis by targeting IL‐31 through its epigenetics modifications
Source: Aging Cell. 2021 Sep 30;20(10):e13485. doi: 10.1111/acel.13485 (PMC8520716; doi:10.1111/acel.13485)
Supplement: Supplementary file 1 — App S1 [file ACEL-20-e13485-s001.docx]

**Appendix S1**

**Supplementary Figures**

**FIGURE S1 miR-195-3p promotes pro-inflammatory cytokine expression in macrophages by IL-31.**

**
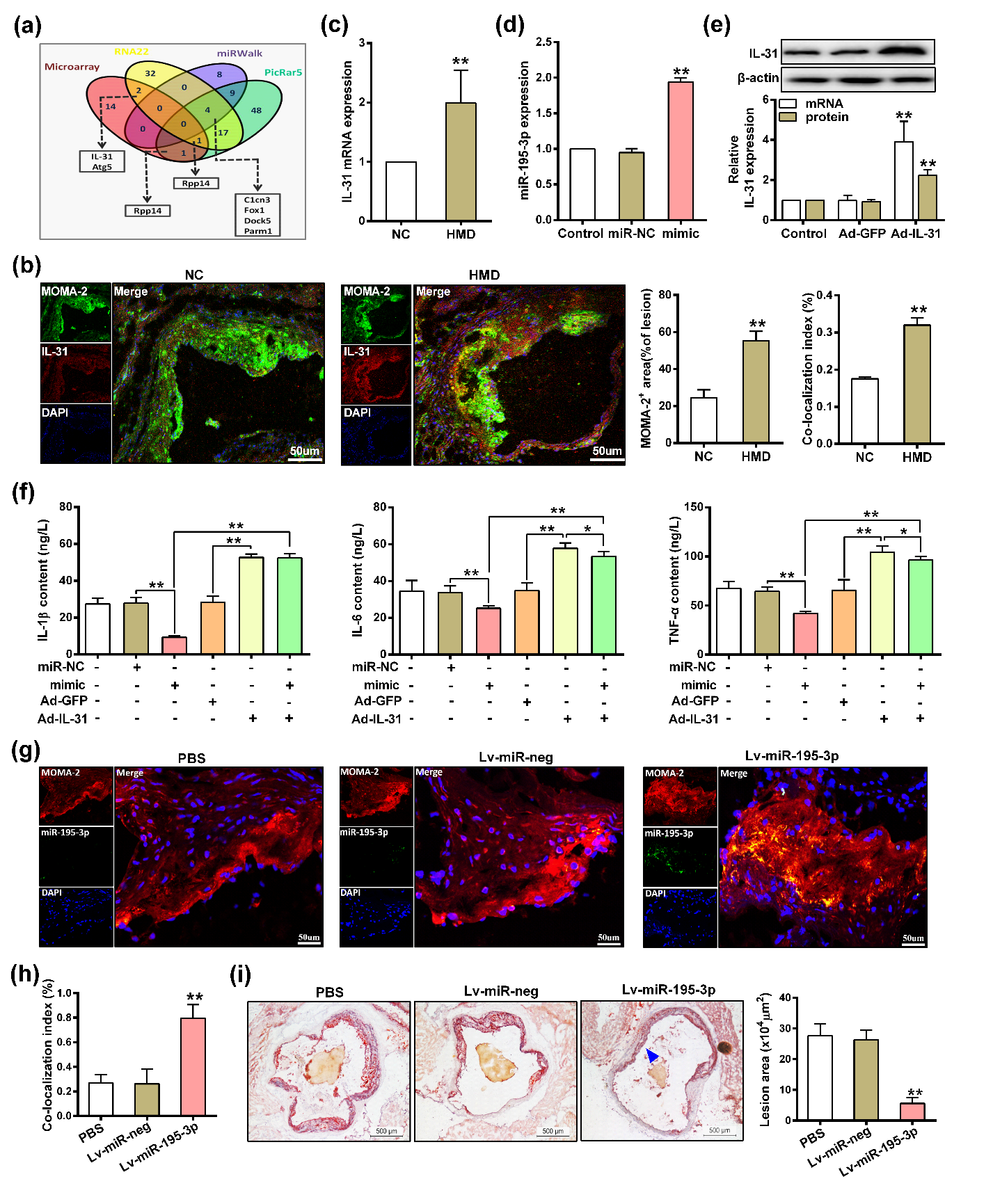
**

**FIGURE S1 miR-195-3p promotes pro-inflammatory cytokine expression in macrophages by IL-31.** **(a)** Microarray analysis and three algorithms (PicTar 5, RNA 22, and miRwalk) were used to predict the miRNA target gene. Venn diagrams show the numbers of genes detected by microarray analysis and three different algorithms, including the numbers of overlapping genes. **(b)** Representative immunofluorescence images and quantification of IL-31 (red) co-localization with MOMA-2 (a marker for macrophages, green) in *ApoE^-/-^* mice. Nuclei were stained with DAPI (blue). Scale bar=50 μm. **(c)** The expression of IL-31 in *ApoE^-/-^* mice were detected by qRT-PCR. **(d)** miR-195-3p expression in macrophages transfected with miRNA negative control (miR-NC) or miR-195-3p mimic was quantified by qRT-PCR. **(e)** The expression of IL-31 in macrophages infected with adenovirus expressing IL-31 (Ad-IL-31) or Ad-GFP were detected by qRT-PCR and western blot. **(f)** The secretion of IL-1β, IL-6 and TNF-α in supernatant of macrophages were detected by ELISA after the cells transfected with miR-NC, miR-195-3p mimic, Ad-IL-31 or Ad-GFP in presence of Hcy. **(g, h)** Representative in situ hybridization of miR-195-3p (green) in atherosclerotic plaques isolated from the HMD-fed *ApoE^-/-^* mice injected with PBS, Lv-miR-neg and Lv-miR-195-3p. Scale bar=50 μm. **(i)** Photomicrographs of aortic root cross-sections and quantitation of plaque area were measured by Oil Red O staining in HMD-fed *ApoE^-/-^* mice injected with PBS, Lv-miR-neg and Lv-miR-195-3p. The blue arrow indicates the plaque region. Scale bar=500 μm. Data were presented as mean ± SD. ^*^*P*<0.05, ^**^*P*<0.01.

**FIGURE S2 The occupancy of histone marks H3K9ac on miR-195-3p promoter.**


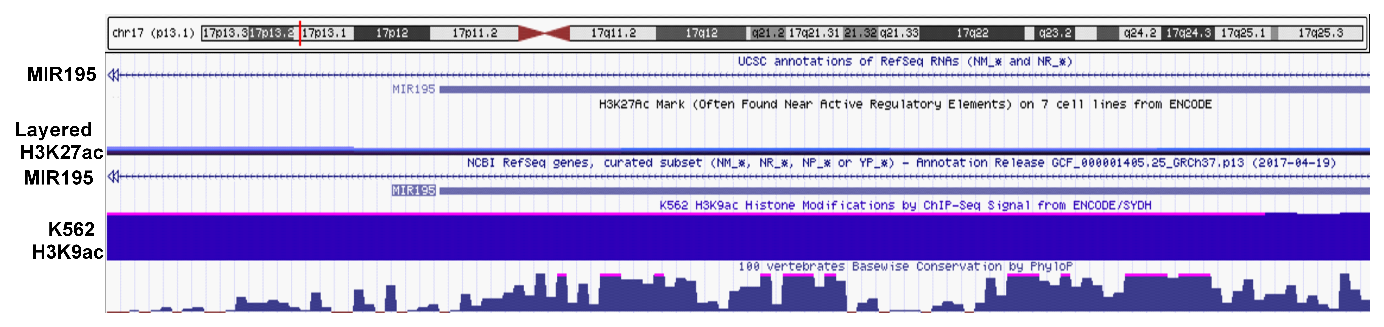


**FIGURE S2 The occupancy of histone marks H3K9ac on miR-195-3p promoter.** UCSC Genome browser tracks showing the enrichment of histone marks H3K9ac and H3K27ac around at miR-195-3p promoter. The top panel represents chromosome 17 with miR-195-3p location indicated by a red vertical line. Bottom panel: miR-195-3p structure with H3K9ac and H3K27ac clusters mapped to the gene.

**FIGURE S3 The expression of key enzymes involved in DNA methylation and histone acetylation.**


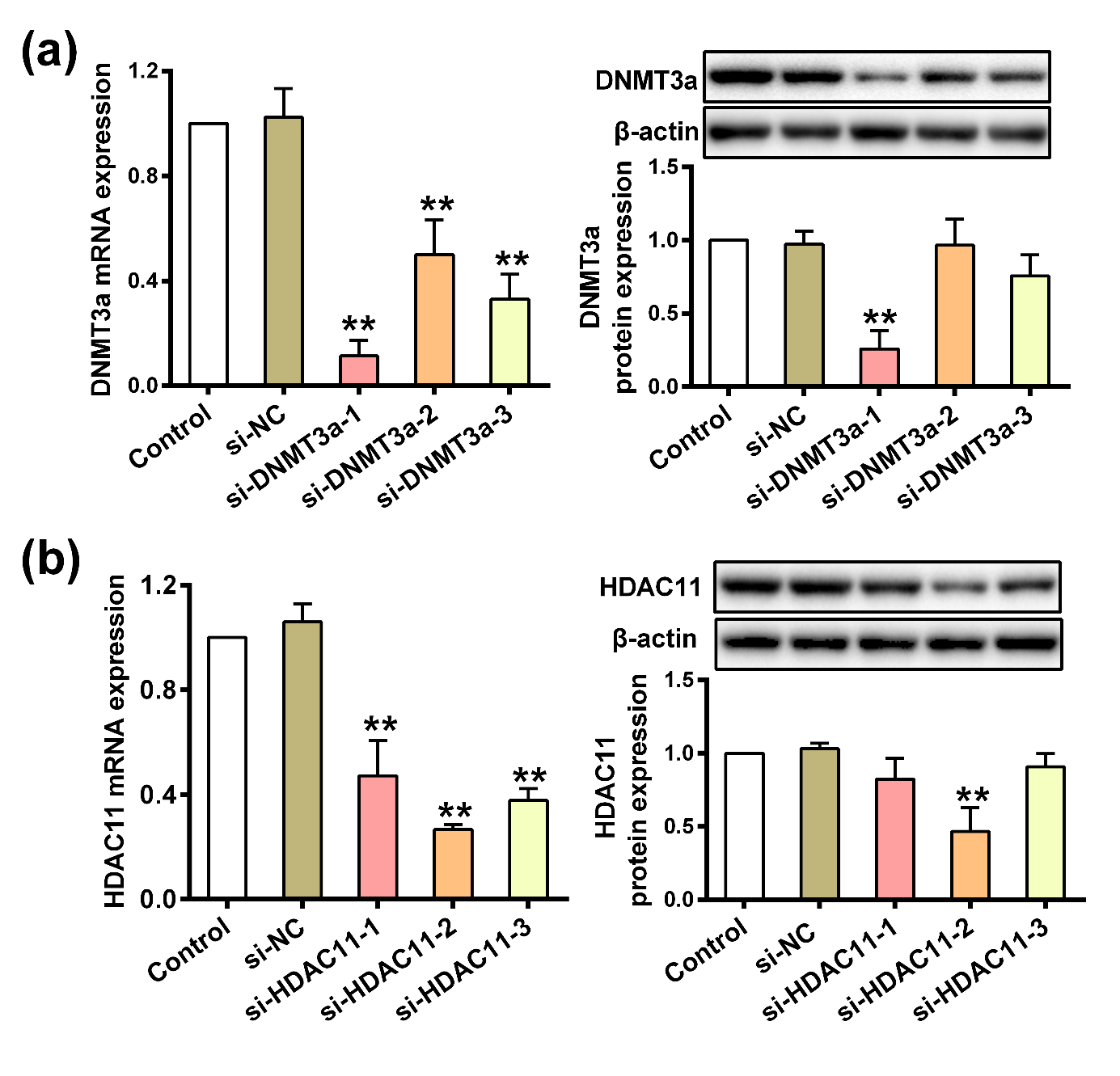


**FIGURE S3 The expression of key enzymes involved in DNA methylation and histone acetylation. (a, b)** Macrophages were transfected with siRNAs against DNMT3a (si-DNMT3a), siRNAs against HDAC11 (si-HDAC11) or negative control siRNA (si-NC), respectively. qRT-PCR and western blot was performed to verify the silence efficiency. Data were presented as mean ± SD. ^**^*P*<0.01.

**FIGURE S4 Overexpression or silence Sp1 in macrophages.**

**
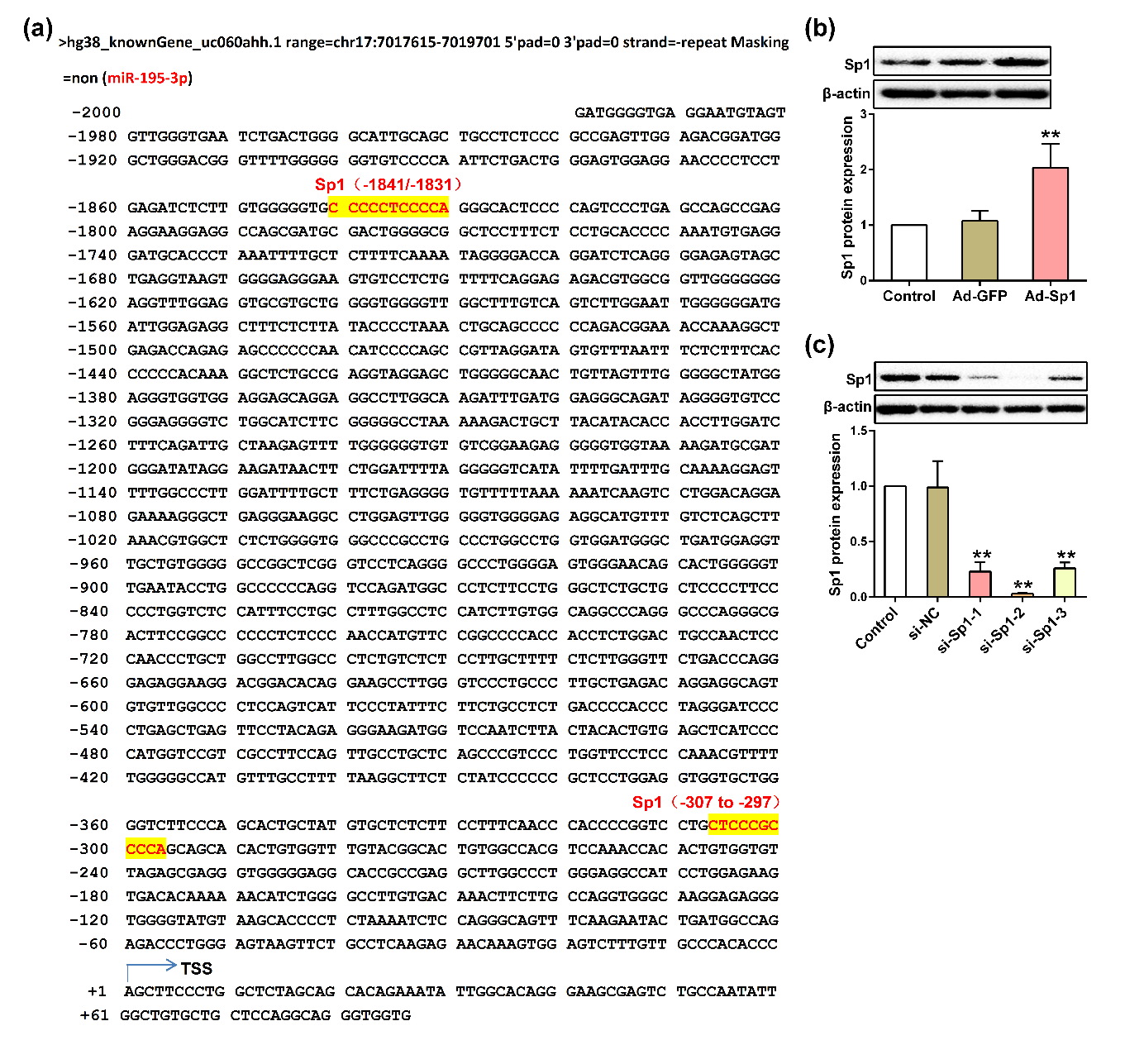
**

**FIGURE S4 Overexpression or silence Sp1 in macrophages.** **(a)** Graphic representation of the putative Sp1 binding sites in the proximal promoter of miR-195-3p. Nucleotide sequence of miR-195-3p proximal promoter was analyzed by Searching Transcription Factor Binding Sites (TFsearch). Two putative binding sites of the Sp1 transcription factor were shadowed by yellow color shown with red in a gray shadow area. miR-195-3p transcription start site (TSS) was indicated by an arrow. **(b, c)** The expression of Sp1 in macrophages infected with adenovirus expressing Sp1 (Ad-Sp1), Ad-GFP, siRNAs against Sp1 (si-Sp1) or control siRNA (si-NC) was detected by western blot. Data were presented as mean ± SD. ^**^*P*<0.01.

**Supplementary Table**

**Table 1. Expression of 12 miRNAs showed difference in the aorta of ApoE^-/-^ mice fed with HMD**

| **Systematic_name** | **p** | **FC (abs)** | **Regulation** |
| --- | --- | --- | --- |
| mmu-miR-144 | 0.011680011 | 5.183816 | up |
| mmu-miR-144* | 8.55846E-05 | 71.40052 | up |
| mmu-miR-149* | 1.87766E-05 | 36.28331 | up |
| mmu-miR-193b | 0.039689705 | 3.277883 | up |
| mmu-miR-3107 | 0.008736538 | 3.341105 | up |
| mmu-miR-34c | 0.04859882 | 66.11395 | up |
| mmu-miR-378 | 0.041095037 | 3.00772 | up |
| mmu-miR-378* | 0.04144196 | 3.992214 | up |
| mmu-miR-378b | 0.0455761 | 3.816116 | up |
| mmu-miR-451 | 0.011462075 | 2.933729 | up |
| mmu-miR-1949 | 0.03904918 | 2.58448 | down |
| **mmu-miR-195-3p** | 0.003840335 | 197.3195 | down |
| mmu-miR-342-5p | 0.000574368 | 33.96591 | down |
| mmu-miR-466c-5p | 0.04782434 | 56.43127 | down |
